# New Hampshire Alliance for Healthy Aging

**DOI:** 10.1093/geroni/igaf122.351

**Published:** 2025-12-31

**Authors:** Jennifer Rabalais

**Affiliations:** University of New Hampshire Institute on Disability, Durham, New Hampshire, United States

## Abstract

The NH Alliance for Healthy Aging (NHAHA) is a statewide collective impact initiative of over 600 stakeholders, representing 300+ organizations (http://nhaha.info/). AHA’s approach is: a) change the conversation about aging; b) change public policy to promote a strong, stable infrastructure for aging; and c) change practices across public and private sectors to improve care and support for older adults, their families, and communities. The work is guided by a shared vision for communities to advance culture, policies, and services which support older adults and their families. Strategic priority areas of NHAHA include: develop and strengthen an advocacy infrastructure to advocate on behalf of the needs of older adults; enhance family caregiver support; improve availability of quality direct care workforce; ensure transportation options are available for older adults across the state; and advance options for diverse housing to allow older adults to remain in their community of choice. . The NH Healthy Aging Data Report continues to support this state level approach by providing data for state plans and other state level initiatives as well as helping communities across the state understand the needs of older adults in their communities.

